# How Can We Assess Positive Welfare in Ruminants?

**DOI:** 10.3390/ani9100758

**Published:** 2019-10-02

**Authors:** Silvana Mattiello, Monica Battini, Giuseppe De Rosa, Fabio Napolitano, Cathy Dwyer

**Affiliations:** 1 Dipartimento di Scienze Agrarie e Ambientali - Produzione, Territorio, Agroenergia, Università degli Studi di Milano, 20133 Milan, Italy; monica.battini@unimi.it; 2 Dipartimento di Agraria, Università degli Studi di Napoli Federico II, 80055 Portici, Italy; giuseppe.derosa@unina.it; 3 Scuola di Scienze Agrarie, Forestali, Alimentari ed Ambientali, Università degli Studi della Basilicata, 85100 Potenza, Italy; fabio.napolitano@unibas.it; 4 Animal Behaviour and Welfare, SRUC, Easter Bush Campus, Edinburgh EH9 3JG, UK; Cathy.Dwyer@sruc.ac.uk

**Keywords:** ruminants, cattle, sheep, goats, buffaloes, animal welfare, positive indicators, five domains

## Abstract

**Simple Summary:**

The concern for better farm animal welfare has been greatly increasing among scientists, veterinarians, farmers, consumers, and the general public over many years. As a consequence, several indicators have been developed to assess animal welfare, and several specific protocols have been proposed for welfare evaluation. Most of the indicators developed so far focus on the negative aspects of animal welfare (e.g., lameness, lesions, diseases, presence of abnormal behaviours, high levels of stress hormones, and many more). However, the lack of negative welfare conditions does not necessarily mean that animals are in good welfare and have a good quality of life. To guarantee high welfare standards, animals should experience positive conditions that allow them to live a life that is really worth living. We reviewed the existing indicators of positive welfare for farmed ruminants and identified some gaps that still require work, especially in the domains of Nutrition and Health, and the need for further refinement of some of the existing indicators.

**Abstract:**

Until now, most research has focused on the development of indicators of negative welfare, and relatively few studies provide information on valid, reliable, and feasible indicators addressing positive aspects of animal welfare. However, a lack of suffering does not guarantee that animals are experiencing a positive welfare state. The aim of the present review is to identify promising valid and reliable animal-based indicators for the assessment of positive welfare that might be included in welfare assessment protocols for ruminants, and to discuss them in the light of the five domains model, highlighting possible gaps to be filled by future research. Based on the existing literature in the main databases, each indicator was evaluated in terms of its validity, reliability, and on-farm feasibility. Some valid indicators were identified, but a lot of the validity evidence is based on their absence when a negative situation is present; furthermore, only a few indicators are available in the domains of Nutrition and Health. Reliability has been seldom addressed. On-farm feasibility could be increased by developing specific sampling strategies and/or relying on the use of video- or automatic-recording devices. In conclusion, several indicators are potentially available (e.g., synchronisation of lying and feeding, coat or fleece condition, qualitative behaviour assessment), but further research is required.

## 1. Introduction

Animal welfare research has led to a better understanding of animal welfare needs and the development of scientific welfare indicators, which have been merged into welfare assessment protocols for various species, including cattle [1], goats [2,3], and sheep [4,5]. These protocols, developed in Europe for the evaluation of animal welfare on farms, include a selection of valid and reliable indicators whose on-farm evaluation is feasible and, whenever possible, follows European Food Safety Authority’s recommendations [6] that indicators are animal-based, whereas resource– and management–based indicators are considered as “risk factors”.

On-farm welfare assessment schemes focus almost exclusively on the evaluation of negative welfare indicators (e.g., lameness, overgrown claws, lesions, abnormal behaviour, excessive aggressiveness, fear) and provide an output on welfare levels based on the quantification of such negative aspects: if the presence of negative indicators is frequent, the level of welfare is low, and vice versa. In this view, the lack of suffering and the satisfaction of animals’ basic requirements is indicative of a good welfare level. This follows the earlier concepts of animal welfare, based on the respect of the Five Freedoms deriving from the Brambell Report [7], formalised by the Farm Animal Welfare Council (FAWC) in 1979, and later revised and translated into the Four Principles and Twelve Criteria during the Welfare Quality^®^ EC Project [1]. These concepts are the drivers of most of the current legislation and codes of practice on animal welfare, essentially based on the avoidance of unnecessary suffering.

However, a lack of suffering does not guarantee that animals are experiencing a positive welfare state. Several studies have argued for the inclusion of positive welfare indicators or consideration of the positive aspects of animal welfare as well as the negative in achieving a more comprehensive view of animal welfare [8]. Animals are motivated to gain a resource or achieve a particular interaction, and the affective state of achieving these goals or the reward is pleasure. Thus, what animals want, in terms of seeking positive or attractive stimuli, are associated with animals “wanting” these rewards [8]. The FAWC [9] suggests that the minimum welfare level should be defined in terms of an animal’s Quality of Life (QoL) over its whole lifetime, and that QoL can range from a very poor level (a life not worth living), to a medium level (a life worth living), up to a high level (a good life).

Given that animal welfare is not only an absence of negative states (e.g., pain, disease, fear), and something positive should be provided to farm animals to make their lives worth living, positive animal welfare remains difficult to define. Previous constructs of animal welfare suggest that welfare can be defined by concerns falling into one of three domains: biological functioning, naturalness, and the feelings of affective experiences of the animal [10]. Following the functional approach, positive welfare may just be something that goes beyond the provision of farming conditions allowing the animals to be in good health. Grazing may be an example: ruminants can survive and be healthy without expressing this behaviour; hence, access to pasture may be considered a benefit and an indicator of positive welfare [11]. However, according to the natural approach, ruminants should be free to express any species-specific behaviour; thus no grazing would be a sign of a negative state, whereas access to pasture would be just the normal condition. The focus could then be on animal feelings, although short-term preferences may not match animal long-term interests. However, according to [12], good welfare can be achieved when animals are able to have a certain degree of control over the surrounding environment while tackling and meeting the challenges. In other words, a good life is not a life without challenges with both too high and too low levels of stimulation possibly perceived as aversive. Animals have expectations about the environment where they live and [13] stated that a positive experience occurs when an animal “actively responds to motivations to engage in rewarding behaviours, including all associated appetitive and consummatory effects that are positive”. For example, if an animal is ‘fully engaged in exploring and food gathering in a stimulus-rich environment and interacts pleasantly with other animals in the social group’, then that animal may be considered to be in a positive welfare state. Both views highlight the active role played by the animals while positively interacting with the surrounding environment.

Therefore, good animal welfare should also be considered in the light of the presence of positive experiences or sensations, and not only as the result of the absence of negative experiences [14], and the balance between positive and negative effects should be in favour of the former [15]. This implies moving from the concept of “Freedoms” towards the concept of “Provisions”, as animals should be managed in order to provide them with a range of opportunities to experience comfort, pleasure, interest, confidence, and a sense of control [15].

In line with these considerations, the OIE Terrestrial Animal Health Code [16] recently stated that “Animal welfare means how an animal is coping with the conditions in which it lives. An animal is in a good state of welfare if (as indicated by scientific evidence) it is healthy, comfortable, well-nourished, safe, able to express innate behaviour, and if it is not suffering from unpleasant states such as pain, fear, and distress”. This definition clearly emphasises the need for positive experiences (health, comfort, good nutrition, and freedom to express natural behaviour) in the first place and mentions the freedom from suffering only at the end.

However, most research has focused on the development of indicators of negative welfare, and relatively few studies provide information on valid, reliable, and feasible indicators addressing positive aspects of animal welfare. The identification of animal-based indicators with these characteristics would allow their inclusion in welfare assessment schemes. This would be beneficial not only for improving the level of animal welfare, leading to a high QoL of farmed animals but also for reinforcing the communication about animal welfare to the stakeholders, who are strongly interested in positive indicators [17].

Mellor [17,18,19] proposes a five domains model to draw attention to important areas deserving consideration when talking about animal welfare. The model takes into consideration four domains related to internal states and external circumstances (i.e., Nutrition, Environment, Health, and Behaviour), and a fifth domain, i.e., Mental State, which is a final component showing positive or negative affective engagement resulting from the sum of internal states and external circumstances.

The aim of the present review is to identify existing valid and reliable animal-based indicators for the assessment of positive welfare that might be included in welfare assessment protocols for ruminants, and to discuss them in the light of the five domains model, highlighting possible gaps to be filled by future research.

## 2. Materials and Methods

As a starting point, an extensive review of scientific literature was carried out in the main databases (Web of Science, CAB Abstracts, PubMed, and Scopus), using keywords such as “positive welfare”, “measure”, “indicator”, “comfort”, “human-animal relationship”, “emotions”, “natural behaviour”, “pleasure”, “liveliness”, “synchronization”, “play” combined with “ruminant”, “cattle”, “cow”, “sheep”, “goat”, and “buffalo”. A total of 45 records, including 12 reviews, were obtained from this initial search. On the basis of the references cited in these records, and of the suggestions from the reviewers of the initial version of this manuscript, we enlarged our search, to obtain the final list included in this review. Only English language studies published in international journals, international book chapters or international protocols were retained. We focused exclusively on animal-based indicators that could be collected on-farm. Animal-based indicators requiring subsequent laboratory analysis were discarded. Publications dealing with resource- and management-based indicators were also excluded.

Based on the existing literature, each indicator was evaluated in terms of its validity, reliability (test–retest reliability, intra- and inter-observer reliability), and on-farm feasibility (Table 1).

Resource- and management-based measures were excluded, and only animal-based measures were considered, as this approach seems more appropriate for measuring the actual welfare state of the animals and the way in which they respond to the farming environment [6].

The results are presented in the light of the five domains considering: Nutrition, Environment, Health, Behaviour, and the overall affective Mental State [17].

Some indicators can provide useful information related to more than one domain; when this was the case, it was specifically mentioned in relation to each domain.

## 3. Promising Indicators in the Five Domains

A list of potential indicators of positive welfare indicators in Mellor’s four domains [17,18,19] related to internal states and external circumstances (i.e., Nutrition, Environment, Health, and Behaviour) is summarised in Table 2.

### 3.1. Nutrition

Positive aspects of welfare associated with the nutrition domain go beyond the bare satisfaction of physiological nutritional requirements, and imply, for example, aspects of choice and variety of food with pleasant smell, taste, and texture, pleasures associated with active engagement and exploration of the environment during foraging, oral pleasures of chewing/sucking, or hedonic properties of food, that eventually lead to a positive mental state [18]. The positive welfare aspects of nutrition would include measures indicative of the hedonic pleasures associated with consuming preferred foods, sensory aspects of pleasurable tastes [57] and the quenching aspects of drinking, as well as the feelings of satiation and the anticipatory pleasures of seeking and consuming food. According to [58], the possibility of food choice provides animals with the freedom to express their normal behaviour, to meet specific individual needs, and also to reduce the incidence of illness by better coping with toxins and parasite loads. Ruminants express feeding preferences if they are allowed to select their diet without any constraints, whereas the diet they actually select represents their preferences as modified by any environmental factors (e.g., accessibility, competition with conspecifics) [59]. Ruminants show feeding preferences based on forage taste, odour, and texture characteristics because these animals can associate such characteristics with the post-ingestion effects of feeds at the gastro-intestinal level [60]. In particular, they are able to avoid unpalatable feeds with low nutrient content or high toxin levels while actively selecting palatable and nutritious feeds with the aim to maximize their nutritional well-being [61]. Catanese et al. [62] confirmed that limiting diet choice induces a stress response in lambs. At least in humans, a close relationship has been found between affect and food consumption (e.g., [63]). Therefore, it has been postulated that also in non-human animals, the ingestion of pleasant feeds, based on their hedonic values, may induce a more positive affective state as compared to receiving less palatable feeds. In fact, [21] observed that after the consumption of a pleasant pellet, ewes show an optimistic judgment bias by approaching non-reinforced ambiguous locations more quickly (i.e., the ewes received no training about the possible presence of palatable or unpalatable feeds in those locations) than ewes receiving disliked wood pellets. These results indicate the expression of feeding preferences as a potentially valid indicator, although no evidence is available on its reliability, and preference tests may not be easily performed on-farm.

In rodents, behavioural indicators of the pleasure of eating or drinking have been quantified as tongue protrusions, lip smacking, and lick patterns (e.g., [64]), but similar animal-based indicators have not been developed for ruminants. Both sheep and cattle consistently show a preference of legumes over grasses (1.5 times higher intake) and also a particular diurnal pattern, with legumes preferred in the morning and preference for grasses increasing at the end of the day [59]. These data suggest that ruminants have specific goals when selecting their diet, which cannot be accomplished when they are fed a total mixed ration, with no possible alternative. However, it should be considered that feed choice may be driven by individual differences [65], as some animals are more prone to consume a regular and constant diet, whereas others are willing to explore new feeds, as recorded in heifers by Meagher et al. [66]. It may be argued that, if animals are given the possibility of choosing their diets, they may not necessarily eat what is best for them, and they may not consume a diet adequate to meet their nutritional requirements. Although we cannot exclude that this may happen in some cases, research by [67] showed that calves offered with a varied diet reached the same nutritional level provided by a standard balanced mixed ration, yet each animal ate a diet different from the other animals. Data supporting the evidence that ruminants are able to select a diet close to their needs and minimise the ingestion of anti-nutritional compounds when they are given the possibility to choose among different feeds are reviewed by [58].

An additional benefit in terms of welfare can be given to ruminant animals by allowing the expression of their dietary preferences while performing their species-specific grazing behaviours (i.e., exploration, selection, and ingestion of plants [22]), as also envisaged by the Welfare Quality protocol [1,12].

Domestic ruminants are gregarious animals and their feeding behaviour, as well as other maintenance behaviours [68,69,70], is synchronised [71]. Feeding behaviour synchronisation in social animals is an adaptive behaviour, evolved to provide a series of benefits, such as opportunities for acquiring information about the location of food and allowing more time to graze, due to reduced exposure to predation risk [72]. In this sense, feeding synchronisation may indicate a positive welfare condition for all group members (see also “Behaviour”), and it was actually observed more frequently in finishing bulls kept on pasture than in a more restricted housing environment [23]. Furthermore, feeding synchronisation may indicate reduced competitiveness, thus representing an additional benefit for subordinate animals, as they would be able to access the feeding resources along with conspecifics. The assessment of the synchrony of feeding is feasible on-farm and it could be achieved by instantaneous scan sampling [68], taking into account that synchronisation is maximal in the morning and in the evening [73]. No information is available about the reliability of feeding synchronisation, but behavioural synchronisation is generally considered reliable [39], whereas the articles mentioned previously [23,39,70,71,72,73] support the validity of the measure.

### 3.2. Environment

The environment can have marked effects on animal welfare, and some literature suggests the positive effects of housing enrichments, which should be beyond the simple housing supplementation (i.e., just capable of reducing the negative effects of a poor environment). Examples of housing enrichment are reviewed by [74,75,76]. Positive aspects of housing or the environment involve providing the animal with the space and requirements for comfort and pleasure associated with resting and ease of movement, as well as offering choice and opportunity to express agency in use of the environment.

Comfort and appropriate rest are important components of positive welfare induced by the environment. For example, reduced lying time and abnormal lying postures or transition movements are shown when housing conditions are suboptimal (e.g., [77,78]). We can, therefore, argue that an increase of lying time, the possibility to perform appropriate lying postures, and the ease to get up and lie down may be indicative of a positive welfare state.

In ruminants (e.g.,: cattle: [34,79]; sheep: [34]), resting is a high priority and an inelastic behavioural need. The total amount of time spent lying was used to assess cow comfort in response to the type of housing (large pens vs tie-stalls) and the depth and shape of sand bedding, respectively [27,28]. In both studies, a higher lying time occurred in the more favourable conditions (large pens and deeper bedding), thus confirming the validity of this positive indicator to assess cow comfort during resting. The time spent lying was also observed to increase in cows, sheep, and in dairy calves provided with a more insulating substrate (e.g., sand bed vs concrete floor in cows [30]; straw bed rather than concrete or slatted flooring in shorn ewes [35]; sawdust rather than river stones in calves [29]), demonstrating lying time to be a good positive indicator for the evaluation of bedding quality and of thermal comfort. Another example that supports the validity of lying time for the evaluation of cows’ comfort in relation to the environment is provided by [31], who observed an increase of the time spent lying on more comfortable lying substrates (i.e., rubber mats vs concrete or sand).

In goats, the total time spent lying was positively affected by increased indoor space allowance [80], but did not statistically vary in response to the inclusion in the pens of additional walls that had been introduced in order to increase goats’ comfort and to provide a higher sense of protection not only from virtual predators, but also from higher-ranking goats. However, goats spent significantly more time resting in the resting area with wall support, suggesting that this indicator (resting by a wall) may be used as an indicator of comfort in this species [32]. In an analogous experiment in sheep, similar results were obtained on lying time, but not on time spent resting by a wall [34]. However, lying time may be increased when animals are unwell (sickness behaviour) or when lame (e.g., [81]); thus, alone, this indicator is not specific for positive welfare.

In cattle, [27] used the frequency of lying bouts as an indicator of the ease of transition movement, and validated this indicator by showing that the number of transition movements was significantly higher in large pens than in tie-stalls. As the mean duration of single lying bouts did not differ between treatments, the higher number of lying bouts resulted in a longer total lying time, which was also considered as a positive indicator of animal welfare. However, [28] could not confirm the validity of the number of lying bouts to assess cows’ comfort, whereas they found significant differences in the duration of lying bouts in relation to bedding depth and shape. In contrast, [33] did not record any significant difference either in total lying time or in the number of lying bouts, in response to different space allowances.

None of the studies mentioned have investigated these indicators in terms of reliability (test-retest, inter-observer, or intra-observer reliability) and the on-farm feasibility of these indicators still has to be discussed. In fact, all the studies were based on either direct- [33] or video-recorded [27,32,34] observations lasting 24 h, which is impractical for on-farm assessment. Alternatively, electronic devices were used in the study by [28], but their use for a practical on-farm assessment is also questionable. Sampling observation rules would be required in order to increase the feasibility of these indicators. However, circadian rhythms of resting periods may show a pronounced variation, especially in extensively managed animals [81], and this may have a marked impact on the selection of sampling periods for the assessment of lying time.

Napolitano et al. [68] suggested that lying postures can also be used to highlight the level of thermal comfort and/or vigilance comfort, and state that cows prefer to rest in sternal recumbency with the head tucked against the flank, and in lateral recumbency, possibly with outstretched legs. A positive correlation was found in dairy cows between cubicle features (i.e., stall width, stall length, amount of straw, area, and type of divider) and some lying postures: stretched forelegs, stretched hindlegs, lying fully stretched [38]. van Erp-van der Kooij et al. [40] confirmed the preference of dairy cows for lying in long and wide postures when at pasture, in a comfortable situation. These authors also carried out a reliability analysis on lying postures at the start of their study, finding an initial moderate agreement (kappa values 0.49 and 0.50) between a trainer and the observers, whereas [39] found good inter-observer reliability and consistency over time for head resting and hind legs stretched. Also, for the observation of lying postures, the development of a representative sampling strategy may be required for practical on-farm observations.

Another important aspect of lying behaviour is the level of synchronisation (see also “Behaviour”), that may be considered as indicative of a positive welfare state. For example, Holstein heifers with larger space allowance exhibited a higher synchronisation of lying behaviour, which was interpreted as a higher welfare level, thus confirming the predictive validity of this indicator [33]. The validity of the synchronisation of resting behaviour could not be confirmed as an indicator of comfort in sheep [34] and goats [32], which did not increase their synchrony after the inclusion of additional walls. However, the presence of additional walls is only one of the factors that can potentially affect lying behaviour: the space available in these studies (1.5 m^2^/head, which corresponds to the minimum recommended value for small ruminants; [69]) may have contributed to the inability of all animals lying down at the same time, independently from the presence of partitions. In fact, [81] observed that sheep are more synchronised when they have more space to lie in an indoor environment. Based on the concept of synchronised lying, [26] proposed the use of a Comfort index, calculated as the number of cows lying in free stalls out of the total number of cows touching a stall. According to these authors, a value greater than 85% is considered a good threshold. The validity of lying synchrony as a positive welfare indicator is confirmed by [38], who found a positive correlation between the maximum synchronous lying and cubicle features (i.e., stall width, stall length, amount of straw, area, and type of divider) providing more space and a more comfortable and insulated lying surface. The same author also found a positive correlation of these features with the percentage of cows ruminating while lying: this indicator can also be considered as indicative of a positive welfare state, as rumination is usually performed by healthy, relaxed, and unstressed cows while lying down [82]. The inter-observer reliability and consistency over time of lying synchrony have been confirmed by [39] both for direct and video-recorded observations.

As for feeding synchrony, also lying synchrony can be assessed using instantaneous scan sampling: this indicator can be collected in a quicker way than lying time, and it is less likely affected by circadian behavioural changes [81].

Another behaviour that may indicate that animals are living in a good environment is exploration, which usually increases in novel and complex environments, as discussed in the paragraph “Behaviour” (e.g., [54]).

### 3.3. Health

Traditionally, assessment of health aspects of welfare has focused on categorising and auditing the most common health issues of the species (number of lame animals, parasitised, showing visible injuries, and so on). However, the absence of clinical disease or injury is not the same as the positive aspects of good health, such as feeling well, active, and vigorous. Suitable positive welfare indicators in this domain would include measures that suggest animals are enjoying vitality and good health.

The longevity of breeding animals can be defined as the time-span animals remain in the herd, whereas productive longevity is the period between first parturition and culling. In intensively farmed dairy cow herds, longevity and productive longevity are usually well below five and three years, respectively [43], whereas cows kept in extensive conditions or in small family farms show a mean longevity of 15 years [83] and cases are reported where animals reach over 22 years [84]. Most of the factors that lead to the culling of dairy cows concern health (e.g., mastitis, lameness, low fertility) and unsatisfactory production. In farm animals, a long life may be considered as the result of good welfare [85], and longevity as its “summary indicator” (i.e., summarising all the potential noxious factors leading to a reduced life expectancy). However, it may be speculated that the validity of this indicator relies on the reliability of the information about culling reasons, as only those concerning involuntary culling should be considered. In addition, there may also be concerns about Quality of Life, as just being on the farm for a long time may not mean positive welfare: a longer time spent in pain but not reaching the point of needing to be culled may have obvious negative effects on the welfare of the animals, and may result in a life that is not “a good life”, and possibly not even “a life worth living” [85].

Vigour is another indicator of positive health, which expresses positive and active engagement with the environment. In lambs, a vigour score has been proposed, based on the latency to first perform specific behaviours, such as an attempt to stand, seeking the udder, and successful sucking [44,45]. This score has been shown to be valid, in that it is reflective of the behaviour of neonatal lambs [45], and has been applied on commercial farms [45] suggesting data can be collected feasibly, at least on farms with indoor lambing systems. The reliability of scoring has not been formally tested.

A positive health condition can also be identified by coat or fleece conditions. For example, the percentage of goats with coat described as “a complete fur cover, even coat, presenting shiny, glossy and sheen hair, homogeneous and well adherent to the body” [42] has been included in the AWIN welfare assessment protocol for goats [3], and a good fleece quality (sufficient fleece, no trailing or over long patches of fleece, no scurf nor lumpiness, nor evidence of ectoparasites) was included in the AWIN welfare assessment protocol for sheep [5]. For hair coat condition in goats, [42] demonstrated high inter-observer reliability, but [86] showed a low consistency over time. For sheep, assessments of fleece quality have good intra-observer, but poor inter-observer reliability, when assessed at a group level [87], but good inter-observer reliability when assessed individually (AWIN 2015, unpublished).

### 3.4. Behaviour

Positive welfare may be evident when animals are able to express active and positive engagement with the environment and in their interactions with other animals, resulting in exploration, foraging, hunting, bonding, affiliative social contacts (such as play, social grooming, and other pleasurable contacts,) and positive parent-offspring interactions [13,88].

Numerous authors report that human and non-human mammals play when they are not exposed to harmful events and threats to fitness, such as abrupt weaning, insufficient nutrient intake [89], disbudding [53], and castration [90]. This may be explained by the fact that this behaviour is not needed for survival; thus, it is not expressed in unfavourable conditions. According to [91], play is self-rewarding, may be included in the behavioural repertoire of the adults albeit at a lower frequency than juveniles of the same species, and it is repetitive, although not stereotypical in form. In addition, play has been associated with positive emotions [92]. Valnickova et al. [93] observed that deprivation of play induced reduced growth in dairy calves, thus suggesting the validity of the absence of this behaviour as an indicator of negative welfare. However, a rebound effect in play is often observed when conditions improve. For example, the play expressed by dairy cows when, during winter housing, they are released into exercise areas, may not be an indication of positive welfare, rather, it may represent a sign of relief from a previous poorer condition [94]. In general, it can be stated that reductions in play are associated with negative affect, whereas there is evidence for an increase in play with positive affect. Anticipatory behaviours can provide information about emotional states and the anticipation of play, being a rewarding event, can be a positive state as shown by an increased frequency of behavioural transitions and duration of walking [95]. However, inferences on anticipatory behaviours should be considered, as long waiting periods may induce frustration [96]. The registration of spontaneous play in young ruminants may not be feasible due to the low level of expression [93]. Three main categories of play have been described: social, locomotor, and object play [97]. Mintline et al. [53] suggested using an arena test to elicit locomotor play and reduce the time needed to record this behavioural expression. These authors noted that the amount of time devoted to locomotor play in the home pen was positively correlated with the time spent playing in the arena test, thus suggesting the validity of the test. However, some open questions remain concerning the feasibility of including this in on-farm assessments, as well as the size and shape of the arena, both affecting the expression of this behaviour, the time elapsing between tests, with more time devoted to play at increasing elapsed times, and the high day–to–day expression variability. No studies on the reliability of this test are currently available.

In young mammals, the possibility of sucking from their dams may also be related to a positive welfare state, as this is a natural and highly motivated behaviour (in the wild, it is essential for survival) [98]. The need for sucking from a teat seems to be confirmed by the fact that, if this natural behaviour is limited or prevented, calves may redirect it towards other targets in the form of abnormal oral behaviour [99], while lambs show a number of behavioural, endocrine, and immune disturbances [100]. Conversely, when young mammals have the opportunity of sucking from their mothers, they perform this rewarding and appeasing behaviour, particularly following behavioural disturbances, suggesting that it has a rewarding and comforting component as well as nutritional. Although valid [22,68,69], to our knowledge, no information is currently available about the reliability of this indicator, and feasibility may be limited due to the time required for the observation of sucking behaviour.

A high level of synchronisation has been mentioned as an indicator of positive welfare in cattle [68], goats [69], and sheep [70]. In fact, this is an allelomimetic behaviour indicative of social cohesion [70]. According to [101], in socially stable groups, 90% of the individuals exhibit the same behaviour at the same time, whereas [73] established a 70% synchrony threshold in cattle at pasture.

The use of the level of behavioural synchronisation (standing, lying, and feeding) in cattle as a measure of positive welfare is supported by earlier research showing that dairy cows’ behaviour is more synchronised on pasture than in tie stalls [41], and later confirmed by [23] in fattening bulls, where the synchronisation was higher in bulls at pasture than in bulls kept in pens in an uninsulated barn. In fact, as recently reviewed by [102], a loss of synchrony may be interpreted as an index of reduced welfare in housed vs pasture-based systems (and vice versa), probably due to the reduction of space allowance, the increased level of disturbance, and the higher competition for lying places.

Stoye et al. [73] observed that the level of synchrony was minimal in the middle of the day and peaked in the morning and in the evening, and suggest that the time of day should, therefore, be taken into account when this variable is used to measure animal welfare. This consideration can be important to set up appropriate sampling rules for behavioural observations and may contribute to increasing the on-farm feasibility of this indicator.

Allogrooming is defined as a licking or grooming behaviour performed between pairs, most commonly on the head, neck, and shoulder [71]. Allogrooming has been widely documented in adult [103]) and juvenile cattle [104], whereas in sheep and goats, it is mainly expressed by mothers to new-born animals [105]. In cattle, this behaviour tends to occur most around the arrival of fresh feed, and in longer bouts at night, with 5 min per day total time spent in allogrooming [46]. Allogrooming is not thought to be related to dominance hierarchies but is thought to be an expression of a close relationship [46,47], relevant for the formation and maintenance of social bonds. Affiliative behaviours have been proposed as indicators of positive welfare [92]. Receipt of allogrooming induces reduced heart rates and half-closed eyes [106]. In addition, it has been observed that the animals which receive more grooming have increased milk production and weight gain [107,108]. However, some issues still need to be resolved with respect to whether higher levels of social licking may be a mechanism to reduce tension [104]. In particular, allogrooming-dependent tension reduction was not seen experimentally [92,109], and higher licking was observed in tethered cattle vs loose cattle [110], perhaps related to the familiarity of neighbours or boredom, and in indoor vs outdoor cows, probably due to proximity [109]. On-farm feasibility of allogrooming seems low as it occurs for a relatively short period per animal, but at higher rates during feeding [46]. However, the feasibility on-farm has not been tested, so methods would need to be developed. Consequently, no studies on test–retest reliability and intra- and inter-observer reliability have been conducted.

Self-grooming is related to a broad behavioural category encompassing licking the coat with the tongue (generally restricted to cattle), rubbing or scratching with teeth (sheep), hind hoof, horns, or against environmental objects (trees, fencing, pen fixtures, etc.), including the use of brushes by dairy cows [111]. Self-grooming has also been documented in goats [112,113]. Studies on innate ‘programmed grooming’ suggest that this behaviour is influenced by age (greater self-grooming in young small ruminant animals; [114]). In wild sheep, this behaviour is related to hygiene as a means of removing dirtiness, ticks, and other ectoparasites, so it may be a motivated behaviour [115]. In general, self-grooming is deemed a comfort activity [116]. Platz et al. [37] found that comfort behaviours of individual hygiene in dairy cattle, such as licking while standing on three legs (licking herself with one leg raised from the floor surface) and caudal licking (licking of caudal parts of the body by concave flexion of the lumbar spine), are only performed on non-slippery flooring. The replacement of concrete slatted flooring by rubber mats may increase self-grooming up to 4-fold. According to [117], self-grooming tends to be performed more often when calves are motivated to express this behaviour, but the environment does not allow to exhibit it. However, whether self-grooming is positive or solely an expression of positive affect is questionable, since self-licking occurs at a higher rate in tie stalls than in loose housed cattle [110], and [118] reports that excessive self-licking can be observed in calves in response to deprivation situations.

In natural environments, cattle scratch and groom themselves on trees or other abrasive surfaces [119]. Therefore, the provision of brushes can be considered as an environmental enrichment [55] that stimulates the animals’ natural behaviour, and brush usage seems to be a ‘luxury’ behaviour with low resilience [120], which therefore may indicate positive affect. According to [121], the voluntary use of cow brushes might be a useful indicator of positive welfare. Using a motivation test, [119] demonstrated that the motivation of cows to access a grooming substrate is as high as their motivation to access fresh food. In calves, choice tests suggest that brushing is perceived as a positive event, but heart rate variability is not affected [56]. Ninomiya [55] showed that beef cattle increase their self-grooming and scratching behaviour when their pen is enriched with a brush, and hypothesises that it is possible that the increased expression of this behaviour could be beneficial in terms of animal health, based on the lower occurrence of liver and intestinal diseases in enriched environments. In addition, brushing facilitates milk let-down and acceptance of milking in heifers, although this result may also be related to habituation to humans rather than to brushing, per se [122]. A complete validation of an indicator is still lacking as well as studies on reliability, but its observation seems feasible on-farm.

Exploration can be distinguished as specific and general, the former directed towards a specific object or event, the latter related to the collection of a broader range of information about the surrounding environment. Exploration has an adaptive value as it is information-gathering about feeding resources, and checking for potential hazards, thus making individuals more prepared for avoiding dangers and finding rapid escapes (e.g., in case of attacks by predators). Exploration seems to be self-rewarding, thus indicating a positive welfare state [52]. General exploration increases when animals are exposed to a novel environment, but it is also performed in known complex environments where this kind of exploration is performed to check for changes. In intensive conditions (i.e., no access to pasture), ruminants tend to extend their periods of inactivity while reducing their exploratory behaviours, thus suggesting a low degree of adaptation to an insufficiently stimulating environment (e.g., [54]). However, the recording of daily exploration is not feasible; thus, [52] proposed a novel object test at the feeding rack as a proxy to estimate the animals’ explorative responses. These authors observed higher levels of specific exploration expressed by bulls kept in a barren environment, but they obtained weak results when this environment was enriched and concluded that the test was not promising. In an arena test, buffalo heifers kept indoors expressed higher levels of specific exploration towards a novel object as compared with animals kept on pasture [123]. The authors hypothesised that animals kept indoors due to the paucity of stimuli were more motivated to perform explorative activities. Therefore, although the arena test is reasonably feasible, its validity as a positive indicator is questionable, and data on reliability are lacking.

Ruminants can be exposed to human contacts, and this happens frequently, especially in dairy animals kept in intensive systems. The quantity and the quality of the human–animal relationship may have a prominent impact on the behaviour, welfare, and productivity of farm animals (e.g., [124]). For example, the use of negative interactions (e.g., shouting, forceful sticking, and slapping) may depress milk production [125], growth rate [126], as well as increase the fear of animals towards humans [127]. On the contrary, positive interactions (e.g., talking quietly, petting, and touching) may have beneficial effects on fertility of dairy cows [128] and growth rate of veal calves [129], provide social comfort [130], induce changes in heart rate and heart rate variability and oxytocin release [131,132], and elicit positive affective states [133]. For cattle, many measures have been proposed to assess the quality of the human–animal relationship, ranging from the observation of both stock people and animal behaviour during routine activity (milking, handling, etc.) [128] to assessing attitudes of stock people towards animals through questionnaires [51,128]. For sheep the AWIN protocol reported a human avoidance test with a familiar person at flock level [5], whereas in cattle the most used indicator has been the avoidance distance of animals to humans, defined as the distance at which an unfamiliar observer is allowed to approach an individual animal before it moves to the side or away [51]. It can be measured either at the feeding place or in the home pen [134], the first being more feasible than the latter. However, all the tests involving a human moving towards the animals may be the results of tolerance and fear, whereas, in the tests where the animals voluntarily approach unfamiliar humans, they actively elicit a contact, which can be interpreted as positive. Examples of tests where the animals approach humans are available in cattle [135] and in goats [3,136], and in responses to separation and reunion in lambs bonded to humans [131]. However, it has been postulated that these tests may be the result of conflicting motivations, such as motivation to explore and fear [135], thus affecting the validity of this indicator. Although feasible, no studies on reliability are available.

In addition to the above-mentioned behaviours that are common to farmed ruminant species, there are some species-specific behaviours whose expression can be considered as an indication of positive welfare. For example, buffaloes are the only domestic ruminants expressing the behaviour of wallowing, which consists of covering the body surface with mud. Therefore, this can be considered a species-specific natural behaviour. Although this behaviour has received little attention, when appropriate facilities are available, buffaloes lie in potholes, ponds, or pools. In particular, previous studies report that from February to July, on average, 31% of the animals were wallowing in the mud [137], whereas this increased to 48% in mid-summer (i.e., from June to August) [54]. Buffaloes have a sparse hair coat and, consequently, a reduced number of sweat glands [138]. Therefore, they use wallowing as a means to efficiently dissipate heat, as also suggested by the higher milk production of buffaloes provided with wallowing facilities [54]. Conversely, when wallowing is denied, buffaloes tend to spend more time idling [137] and lying in the slurry (possibly trying to compensate for the lack of water) and less on exploration [54]. In addition, wallowing proved to be more efficient in heat dissipation than showers [139]. However, all these findings just suggest that buffaloes may suffer if wallowing is denied, whereas to be positive, such behaviour should be self-rewarding. One aspect suggesting this effect is that, albeit less frequently, buffaloes wallow also in winter, when a thermoregulatory motivation is lacking [137]. In addition, at least in pigs, wallowing may induce relaxation [140]. This indicator may be considered both feasible and reliable if assessed as a resource (i.e., access to pools/potholes), whereas no information is available if assessed as an expression of wallowing behaviour.

Goats are known to climb and prefer elevated places [141]. Aschwanden et al. [142] found that the provision of platforms to loose-housed goats positively affects the behaviour of animals during feeding and resting and reduces aggressions. The authors found an increase in feeding and resting bouts, while the possibility to move both in horizontal and vertical space helped to minimise agonistic interactions [142]. When given the possibility, goats actively used enriched environments (e.g., niches, platforms, brushes) and were frequently observed climbing, walking, and lying in elevated places [25]. Observations of behaviours influenced by the presence of platforms or elevated places are time-consuming; hence, feasibility is presently low. No information is given about the reliability of this indicator.

One of the most important social interactions animals engage in are the contacts between mothers and offspring. In sheep and beef cattle, where offspring remain with the mother for prolonged periods, affiliative social contacts (licking, nosing, actively and preferentially seeking one another, and lying in contact) are frequent (e.g., [143,144]), and associated with elevated oxytocin in the mother (Muir & Dwyer [145]). Maternal care can be assessed by measuring grooming, mother-offspring contact and proximity, and suckling frequency. At present, these are time-consuming and impractical in the field, but with technological developments, such as proximity sensors, these may become more feasible measures in the future.

### 3.5. Mental State

According to Mellor [20], this domain is the result of internal states and external circumstances that may affect welfare-relevant mental experiences. However, the aim of this review is to consider indicators that can measure the positive mental experience, as generated by other factors. The indicators included in this section are not necessarily linked to a specific provision to the animals but can serve as a general overview of the affective state of ruminants. Positive emotional states of relevance to animal welfare may include, for example, calmness, relaxation, curiosity, excitement, positive engagement, and anticipation of reward or pleasurable events. Panksepp [146] postulated that affective states are functional for the fitness of the animals as positive states inform the animals about the fact that they are coping well, whereas negative states may alert subjects to potential threats to survival.

Based on previous studies of humans, [147] postulated that animals experiencing negative emotions, such as those deriving from fear, stress, and adverse environmental conditions, will interpret ambiguous stimuli in a more pessimistic way [148,149]. Conversely, albeit less studied, more optimistic judgments are given as a consequence of positive experiences such as gentle handling [150], environmental enrichment [151], and release after an aversive treatment [152]. In particular, Doyle et al. [152] noted that ten Merino sheep exhibited a positive bias when released after a period of restraint and isolation. Similar results were obtained by Sanger et al. [153]. These authors observed that shorn sheep had a more positive judgment of ambiguous stimuli after release. These studies showed a more positive response when animals were released from a negative experience, which is assumed to generate a positive affective state, although whether this is also true when there is no prior negative stimulus is yet to be tested. Although a number of physiological measures demonstrate the validity of positive judgement bias as positive welfare indicator, which directly reflects the emotional state of the animal (core affect), on-farm feasibility of this indicator seems low due to the fact that animals have to be trained and specific and articulated tests requiring a specific set-up, rather than the observation of spontaneous behaviour in an undisturbed environment, have to be put in place. Attention bias, another class of cognitive bias, has been developed in order to improve the feasibility of measuring these cognitive effects on-farm. Attention bias describes the differential allocation of attentional resources towards one stimulus compared to others [154]. Recently, [155] proposed the attention bias test where the animals do not need to be trained as the attention towards a novel, potentially noxious stimulus is recorded the first time they encounter it. These authors noted reduced attention towards a dog in more relaxed sheep (i.e., treated with diazepam). However, in cattle, this effect was not replicated [156]. No studies on intra- and inter-observer reliability have been conducted.

Behavioural laterality has also been linked to either positive or negative animals’ emotions, as the two brain hemispheres control contrasting emotions: in fact, the left hemisphere is believed to control positive emotions, whereas the right hemisphere controls negative ones, and behavioural responses are contralateral to the dominating hemisphere [157]. In ruminants, behavioural lateralisation in response to different emotion-eliciting situations has been observed in sheep that showed a higher proportion of left-lateralised ears after the exposure to a non-palatable food (wooden pellet), and a lower proportion of left-lateralised ears after the exposure to a standard feed that was considered as a positive stimulus [158]. A higher proportion of right-lateralised ears can, therefore, be supposed to indicate a positive emotional state. However, the results of this experiment were not consistent with other trials carried out by the same authors, and the validity of ear lateralisation as an indicator of sheep’s emotions still has to be confirmed. This indicator seems to be feasible on-farm, but no information is available on its reliability.

Some indicators may be used to interpret emotions according to a dimensional theory [159] where emotions are described as moving in a continuum along two axes: valence that expresses positive or negative moods, and arousal that defines the low or high level of excitement. Negative emotions often go along with high arousal and positive emotions with low arousal, but high arousal can sometimes also be found in positive situations. The qualitative assessment of animal behaviour (QBA) has been widely used to describe how animals interact with the environment. It is based on an integrative approach where the ‘whole-animal’ is assessed, according to the above-mentioned dimensional theory. This methodology relies on the use of behavioural descriptors ranging from low (e.g., calm, relaxed) to high levels of arousal (e.g., active, restless) and from a positive (e.g., curious, excited) to a negative valence/mood (e.g., indifferent, bored). These descriptors, in the original version, were generated by the observers using the free choice profile technique [160]. The approach based on free choice profile showed that QBA had high intra-observer reliability, inter-observer reliability (e.g., [160]) and validity (e.g., [161]). However, in order to make the methodology suitable for on-farm welfare assessment, fixed lists of terms have also been used and show good inter-observer reliability in cattle [162] and sheep [163]; test-retest and intra-observer reliability, as well as on-farm feasibility, have also been confirmed in sheep [164]. The validity of QBA to highlight positive emotional states has been confirmed in cattle [165], goats [166], and sheep [164].

Farm animals may also express emotions using a complex set of facial expressions, body postures, and vocalisations. Most studies focused on the assessment of these indicators in relation to pain or fear, but they are rarely adopted to assess positive emotions, such as relaxation or pleasure. Behavioural studies and the physiological basis of changes in visible eye white (or eye aperture) suggest that this indicator may reflect emotional experience in cows [167,168,169,170,171] and sheep [172,173,174,175]. In particular, changes in eye aperture can be a dynamic indicator of emotional states, with a low percentage of visible white indicating satisfaction and low arousal [168]. The percentage of eye white decreases when cow and calf are reunited after separation [170], sheep brushed by a familiar human show a high proportion of closed and half-closed eyes during and post brushing, indicating that this procedure might have elicited a relaxed state [172,173], and groomed sheep show low relative eye aperture [175]. However, the percentage of eye white in dairy cows may also increase in response to a positive stimulus presumed to be particularly exciting [171], including exposure to concentrate [169]. Hence, the visible eye white may indicate arousal perhaps more than valence. Validity is uncertain in several studies [169,171,174], whereas inter-observer reliability has been only tested and checked in two studies [169,171]. Reefmann et al. [175] comment on feasibility, and state that determining the eye aperture and, consequently, visible eye white, is a labour-intensive task, made of a digital calculation of the white area in comparison with the whole eye. At present, feasibility is very low for on-farm assessment; however, it could be improved by performing direct observations or developing an automatic computerised calculation. A recent work [176] checked the possibility of assessing the eye white in dairy cows as classes of eye aperture, ranging from eye white clearly visible to half-closed eye: this method appears to be promising and more feasible than any computerised calculation.

Ruminants have highly developed muscles around their ears, enabling them to independently move forward and backwards very readily to express internal states and communicate [158]. Some studies support the idea that facial expressions can communicate specific emotional states, by showing that goats and sheep avoid images of conspecific faces demonstrating fear or discomfort, and are attracted to relaxed or positive facial expressions [177]. Ear postures are highly species-depending and require specific study in order to gather accurate information [178]. Hanging ears in dairy cows and sheep are associated with positive emotional states of low arousal (e.g., stroking or grooming [131,172,173,178,179]) and related to the relaxed/calm dimension of QBA [130,178]; however, whether hanging ears would be associated with a low arousal negative state (such as boredom) has not been rigorously tested. Both backwards and forwards ears may be associated either with positive or negative situations [158,178,180,181]; hence, it is not possible to accurately relate these to underlying emotional states. Frequent ear changes are associated with negative stimuli in sheep [158], but with positive stimuli in cows [178]. Further studies are needed to clarify the meaning of ear-posture changes for each species, and to check for the reliability of this indicator.

Recent research focusing on body posture as an indicator of emotion in farm animals shows that there is considerable variation among species in the meaning of the same posture [182]. Hence, body postures cannot be generalised to different species and, furthermore, it is suggested that specific postures may have a different meaning when assessed alone or combined to a whole body posture evaluation [182]. Stretching the neck, or a horizontal neck position are associated with positive emotions in cows [167,179,182], as well as tail wagging in cattle [182] and sheep [173] and tail up in goats [181]. These postures are usually associated with ears hanging down and can be easily recorded on-farm, but no specific study is available on their reliability.

Vocalisations could be considered as a direct expression of emotion in animals [92]. Little work is available to reliably identify types of calls or acoustic parameters when ruminants are exposed to positive situations; thus, general conclusions are difficult to draw. The source–filter theory of voice production suggests that vocalisations in mammals are generated by vibrations of the vocal folds (“source”) that determines the frequency (or pitch; “F0”), subsequently filtered by the vocal tract (“filter”). Hence, changes in vocal production are associated with emotion-related changes in the pharynx and glottis which alter the characteristics of the sound produced [183]. Low-frequency calls have been recorded in cows, sheep, and goats in situations that elicit positive emotions [181,183,184]. Cows and sheep produce these calls with the mouth closed or partially open [183]. It has also been found that in goats, the vocal fold vibrates at a more stable rate during positive than negative emotions, resulting in more stable F0 over time [181]. However, the use of vocalisations as an indicator to measure positive emotions has some limitations. First, it relies on animals providing enough vocalisations for assessment, and then, on the ability of human assessors to evaluate sounds. The development of automatic tools for the recognition of sound would be particularly appealing to improve the feasibility and reliability of this indicator.

A list of indicators of affective engagement resulting from the sum of internal states and external circumstances (i.e., Mental State) is presented in Table 3.

## 4. Conclusions

The present review allowed the identification of a list of promising indicators that might be included in welfare assessment protocols for ruminants. Most of them cover several aspects of positive welfare related to three domains: Environment, Behaviour, and Mental State. Few positive welfare indicators are available for the evaluation of Nutrition and Health. Thus, further research is needed for these last domains.

Many indicators can be considered valid to highlight positive welfare conditions. However, much of the validity evidence is based on their absence when a negative situation is present; in order to affirm their validity as indicators of positive welfare, it would, therefore, be important to validate them also in the opposite direction, demonstrating their increase in pleasurable situations. This should be a relevant topic for future research. Reliability also needs to be further investigated, as very few studies focus on this aspect; however, in the very few cases where it has been tested, the results seem to be good.

Some indicators are apparently feasible on-farm, but most of them require the development of specific sampling strategies and/or rely on the use of video- or automatic-recording devices. Automation and the use of machine learning systems would help to increase feasibility.

In conclusion, several indicators are potentially already available (e.g., synchronisation of lying and feeding, coat or fleece condition, QBA), but further indicators are required for some domains, and some further testing and refinement is needed for those that are already available. Filling these gaps would be extremely useful in order to set up new welfare assessment protocols able to focus on indicators of positive welfare, in order to assure a higher quality of animal products to consumers, with the guarantee that animals are really experiencing a life that is worth living.

## Figures and Tables

**Table 1:** Definitions of terms proposed by Battini et al. [20] and used in the present review to describe the characteristics of the considered indicators.

Term	Definition
Validity	The relation between a variable and what it is supposed to measure or predict. It can be shown by the ability of an indicator to predict some later criterion, such as a state of pleasure, comfort, vitality, etc. (predictive validity), or by the correlation between an indicator and other measures to which it is theoretically related (i.e., gold standard) (concurrent validity)
Reliability	The extent to which a measurement is repeatable and consistent
Test–retest reliability	The extent to which a measurement is repeatable and consistent throughout time
Intra-observer reliability	The agreement between successive observations of the same individual or group by a single observer, based on statistical significance of correlations (*p* < 0.05) or to Kendall’s coefficient of concordance (>0.7). According to time between measurements, reliability may be classified in short- (1–7 days), medium- (1 week to 1 month), or long-term reliability (>1 month)
Inter-observer reliability	The agreement between different observers during a simultaneous observation, based on statistical significance of correlations (*p* < 0.05) or to Kendall’s coefficient of concordance (>0.7)
On-farm feasibility	The practical chance of using the indicators during on-farm inspection. It may consider different constraints, e.g., time, cost, accessibility, equipment requirements, no laboratory analysis

**Table 2:** List of the reviewed potential positive welfare indicators related to internal states and external circumstances (i.e., Nutrition, Environment, Health, and Behaviour). The animal category and method used for data collection are also specified for each indicator.

Provisions	Welfare Indicator	Animal Category	Data Collection Method	References
	Expression of feeding preferences	Sheep	Direct observations	[21]
	Grazing behaviour	Beef cattle	Direct observations	[22]
	Synchronisation of feeding	Beef cattle	Direct observations	[23]
Environment	Bipedal stance	Goat kid	Direct observations	[24]
	Climbing	Goat	Video recording	[25]
	Comfort index	Dairy cow	Video recording	[26]
	Duration of lying bouts	Dairy cow	Video recording	[27]
		Dairy cow	Electronic device	[28]
	Duration of lying time	Calves	Video recording	[29]
		Dairy cow	Video recording	[27]
		Dairy cow	Electronic device	[28]
		Dairy cow	Video recording	[30]
		Dairy cow	Video recording	[31]
		Goat	Video recording	[32]
		Heifer	Direct observations	[33]
		Sheep	Video recording	[34]
		Sheep	Video recording	[35]
	Exploration/chewing of branches	Goat	Direct observations	[36]
	Frequency of lying bouts	Dairy cow	Video recording	[27]
		Dairy cow	Electronic device	[28]
		Heifer	Direct observations	[33]
	Licking while standing on 3 legs	Dairy cow	Video recording	[37]
	Lying posture (sternal recumbency with head against the flank, in lateral recumbency with stretched legs, lying fully stretched)	Dairy cow	Direct observations	[38]
		Dairy cow	Direct observations	[39]
		Dairy cow	Direct observations	[40]
	Nibbling on objects	Goat	Video recording	[25]
	Playing	Goat	Direct observations	[36]
		Goat kid	Direct observations	[24]
	Ruminating while lying	Dairy cow	Direct observations	[40]
	Step up on an object	Goat kid	Direct observations	[24]
	Synchronisation of lying	Dairy cow	Direct observations	[41]
		Goat	Video recording	[32]
		Heifer	Direct observations	[33]
		Sheep	Video recording	[34]
	Time lying by a wall	Goat	Video recording	[32]
		Sheep	Video recording	[33]
	Use of brush	Goat	Video recording	[25]
Health	Fleece quality	Sheep		[3]
	Hair coat condition	Dairy goats	Direct observations	[42]
	Months staying in the herd	Dairy cow	Direct observations	[43]
	Vigour score	Lambs	Direct observations	[44,45]
Behaviour	Allogrooming	Dairy cow	Video recording	[46]
		Dairy cow	Video recording	[47]
	Avoidance distance at feeding place	Beef cattle	Direct observations	[48]
		Buffalo	Video recording	[49]
		Dairy cow	Direct observations	[50]
	Avoidance distance in the barn	Dairy cow	Direct observations	[51]
	Exploration	Beef cattle	Direct observations	[52]
	Licking while standing on 3 legs	Dairy cow	Video recording	[37]
	Locomotor play	Veal calf	Video recording	[53]
	Percentage of animals in the mud	Buffalo	Direct observations	[54]
	Self-grooming	Beef cattle	Direct observations	[55]
		Veal calf	Video recording	[56]
	Synchronisation of behaviours	Dairy cow	Direct observations	[41]
		Beef cattle	Direct observations	[23]

**Table 3:** List of the reviewed potential positive welfare indicators of affective engagement resulting from the sum of internal states and external circumstances (i.e., Mental State). The animal category and method used for data collection are also specified for each indicator.

Welfare Indicator	Animal Category	Data Collection Method	References
Asymmetric ear posture	Sheep	Video recording	[173]
Axial/plane ears	Sheep	Video recording	[175]
Body posture changes	Sheep	Video recording	[173]
Closed eyes	Sheep	Video recording	[173]
Duration in each ear posture	Sheep	Video recording	[172]
Ear-posture changes	Sheep	Video recording	[172]
Ears back down	Dairy cow	Video recording	[182]
Ears back up	Dairy cow	Video recording	[182]
Ears backwards	Dairy cow	Video recording	[178]
	Dairy cow	Photos	[176]
	Sheep	Video recording	[172]
Ears hanging	Dairy cow	Video recording	[178]
	Dairy cow	Video recording	[179]
	Dairy cow	Photos	[176]
	Lamb	Video recording	[131]
	Sheep	Video recording	[158]
Half-closed eyes	Dairy cow	Photos	[176]
	Sheep	Video recording	[172]
	Sheep	Video recording	[173]
Head orientation changes	Sheep	Video recording	[173]
Infrequent ear-changes	Sheep	Video recording	[174]
Leaning into stroker	Dairy cow	Video recording	[167]
Licking stroker	Dairy cow	Video recording	[167]
Low percentage of visible eye white	Dairy cow	Video recording	[167]
	Dairy cow	Video recording	[169]
	Dairy cow	Video recording	[170]
	Dairy cow	Video recording	[168]
	Dairy cow	Video recording	[171]
	Dairy cow	Photos	[176]
	Sheep	Video recording	[174]
Low relative eye aperture	Sheep	Video recording	[175]
	Dairy cow	Photos	[176]
Low-frequency calls	Dairy cow	Electronic device	[183]
	Goat	Video recording	[181]
Neck horizontal	Dairy cow	Video recording	[182]
Neck stretching	Dairy cow	Video recording	[167]
	Dairy cow	Video recording	[179]
Positive bias	Sheep	Direct observations	[152]
Proportion of right-lateralised ears	Sheep	Direct observations	[158]
Qualitative Behaviour Assessment	Beef cattle	Direct observations	[162]
	Beef cattle	Direct observations	[165]
	Dairy cow	Direct observations	[162]
	Goat	Direct observations	[166]
	Sheep	Video recording	[163]
	Sheep	Direct observations	[164]
	Veal calf	Direct observations	[162]
Rubbing stroker	Dairy cow	Video recording	[167]
Ruminating	Sheep	Video recording	[173]
Sniffing stroker	Dairy cow	Video recording	[167]
Tail up	Goat	Video recording	[181]
Tail wagging	Sheep	Video recording	[173]
Total duration of tail wagging	Sheep	Video recording	[172]
Vigorous tail wagging	Dairy cow	Video recording	[182]

## References

[1] Welfare Quality Consortium (2009). Welfare Quality^®^ Assessment Protocol for Cattle.

[2] Battini M., Stilwell G., Vieira A., Barbieri S., Canali E., Mattiello S. (2015). On-farm welfare assessment protocol for adult dairy goats in intensive production systems. Animals.

[3] AWIN (2015). AWIN Welfare Assessment Protocol for Goats.

[4] Caroprese M., Napolitano F., Mattiello S., Fthenakis G.C., Ribó O., Sevi A. (2016). On-farm welfare monitoring of small ruminants. Small Rumin. Res..

[5] AWIN (2015). AWIN Welfare Assessment Protocol for Sheep.

[6] EFSA (2012). Statement on the use of animal-based measures to assess the welfare of animals. EFSA J..

[7] Brambell Report (1965). Report of the Technical Committee to Enquire into the Welfare of Animal Kept under Intensive Livestock Husbandry Systems.

[8] Yeates J.W., Main D.C.J. (2008). Assessment of positive welfare: A review. Vet. J..

[9] Farm Animal Welfare Council (2009). Farm Animal Welfare in Great Britain: Past, Present and Future.

[10] Fraser D. (2008). Understanding animal welfare. Acta Vet. Scand..

[11] Burow E., Rousing T., Thomsen P.T., Otten N.D., Sorensen J.T. (2013). Effect of grazing on the cow welfare of dairy herds evaluated by a multidimensional welfare index. Animal.

[12] Sachser N., Broom D.M. (2001). What is important to achieve good welfare in animals?. Dahlem Workshop Report 87—Coping with Challenge—Welfare in Animals Including Humans.

[13] Mellor D.J. (2015). Enhancing animal welfare by creating opportunities for positive affective engagement. N. Z. Vet. J..

[14] Green T.C., Mellor D.J. (2011). Extending ideas about animal welfare assessment to include “quality of life” and related concepts. N. Z. Vet. J..

[15] Mellor D.J. (2016). Updating animalwelfare thinking: Moving beyond the “five freedoms” towards “A lifeworth living”. Animals.

[16] OIE (2019). Introduction to the recommendations for animal welfare. Terr. Anim. Heal. Code.

[17] Vigors B. (2019). Citizens’ and Farmers’ Framing of ‘Positive Animal Welfare’ and the Implications for Framing Positive Welfare in Communication. Animals.

[18] Mellor D.J. (2017). Operational details of the five domains model and its key applications to the assessment and management of animal welfare. Animals.

[19] Mellor D.J., Beausoleil N.J. (2015). Extending the “Five Domains” model for animal welfare assessment to incorporate positive welfare states. Anim. Welf..

[20] Battini M., Vieira A., Barbieri S., Ajuda I., Stilwell G., Mattiello S. (2014). Invited review: Animal-based indicators for on-farm welfare assessment for dairy goats. J. Dairy Sci..

[21] Verbeek E., Ferguson D., Quinquet de Monjour P., Lee C. (2014). Generating positive affective states in sheep: The influence of food rewards and opioid administration. Appl. Anim. Behav. Sci..

[22] Kilgour R.J., Uetake K., Ishiwata T., Melville G.J. (2012). The behaviour of beef cattle at pasture. Appl. Anim. Behav. Sci..

[23] Tuomisto L., Huuskonen A., Jauhiainen L., Mononen J. (2019). Finishing bulls have more synchronised behaviour in pastures than in pens. Appl. Anim. Behav. Sci..

[24] Tölü C., Göktürk S., Savaş T. (2016). Effects of weaning and spatial enrichment on behavior of Turkish saanen goat kids. Asian Australas. J. Anim. Sci..

[25] Stachowicz J., Gygax L., Hillmann E., Wechsler B., Keil N.M. (2018). Dairy goats use outdoor runs of high quality more regardless of the quality of indoor housing. Appl. Anim. Behav. Sci..

[26] Overton M.W., Moore D.A., Sischo W.M., Janni K. (2003). Comparison of commonly used ndices to evaluate dairy cattle lying behavior. Proceedings of the Fifth International Dairy Housing Proceedings.

[27] Haley D.B., Rushen J., de Passillé A.M. (2010). Behavioural indicators of cow comfort: Activity and resting behaviour of dairy cows in two types of housing. Can. J. Anim. Sci..

[28] Drissler M., Gaworski M., Tucker C.B., Weary D.M. (2010). Freestall Maintenance: Effects on Lying Behavior of Dairy Cattle. J. Dairy Sci..

[29] Sutherland M.A., Stewart M., Schütz K.E. (2013). Effects of two substrate types on the behaviour, cleanliness and thermoregulation of dairy calves. Appl. Anim. Behav. Sci..

[30] Sahu D., Mandal D.K., Hussain Dar A., Podder M., Gupta A. (2019). Modification in housing system affects the behavior and welfare of dairy Jersey crossbred cows in different seasons. Biol. Rhythm Res..

[31] Norring M., Manninen E., de Passillé A.M., Rushen J., Saloniemi H. (2010). Preferences of dairy cows for three stall surface materials with small amounts of bedding. J. Dairy Sci..

[32] Ehrlenbruch R., Jørgensen G.H.M., Andersen I.L., Bøe K.E. (2010). Provision of additional walls in the resting area—The effects on resting behaviour and social interactions in goats. Appl. Anim. Behav. Sci..

[33] Nielsen L.H., Mogensen L., Krohn C., Hindhede J., Sørensen J.T. (1997). Resting and social behaviour of dairy heifers housed in slatted floor pens with different sized bedded lying areas. Appl. Anim. Behav. Sci..

[34] Jørgensen G.H.M., Andersen I.L., Bøe K.E. (2009). The effect of different pen partition configurations on the behaviour of sheep. Appl. Anim. Behav. Sci..

[35] Færevik G., Andersen I.L., Bøe K.E. (2005). Preferences of sheep for different types of pen flooring. Appl. Anim. Behav. Sci..

[36] Bøe K.E., Ehrlenbruch R., Andersen I.L. (2012). Outside enclosure and additional enrichment for dairy goats—A preliminary study. Acta Vet. Scand..

[37] Platz S., Ahrens F., Bendel J., Meyer H.H.D., Erhard M.H. (2008). What Happens with Cow Behavior When Replacing Concrete Slatted Floor by Rubber Coating: A Case Study. J. Dairy Sci..

[38] Hörning B. (2003). Attempts to integrate different parameters into an overall picture of animal welfare using investigations in dairy loose houses as an example. Anim. Welf..

[39] Plesch G., Broerkens N., Laister S., Winckler C., Knierim U. (2010). Reliability and feasibility of selected measures concerning resting behaviour for the on-farm welfare assessment in dairy cows. Appl. Anim. Behav. Sci..

[40] Van Erp-van der Kooij E., Almalik O., Cavestany D., Roelofs J., van Eerdenburg F. (2019). Lying Postures of Dairy Cows in Cubicles and on Pasture. Animals.

[41] Krohn C.C., Munksgaard L., Jonasen B. (1992). Behaviour of dairy cows kept in extensive (loose housing/pasture) or intensive (tie stall) environments. I. Experimental procedures, facilities, time budgets-diurnal and seasonal conditions. Appl. Anim. Behav. Sci..

[42] Battini M., Peric T., Ajuda I., Vieira A., Grosso L., Barbieri S., Stilwell G., Prandi A., Comin A., Tubaro F. (2015). Hair coat condition: A valid and reliable indicator for on-farm welfare assessment in adult dairy goats. Small Rumin. Res..

[43] De Vries A. (2017). Economic trade-offs between genetic improvement and longevity in dairy cattle. J. Dairy Sci..

[44] Matheson S.M., Rooke J.A., McIlvaney K., Jack M., Ison S., Bnger L., Dwyer C.M. (2011). Development and validation of on-farm behavioural scoring systems to assess birth assistance and lamb vigour. Animal.

[45] Matheson S.M., Bünger L., Dwyer C.M. (2012). Genetic parameters for fitness and neonatal behavior traits in sheep. Behav. Genet..

[46] Val-Laillet D., Guesdon V., von Keyserlingk M.A.G., de Passillé A.M., Rushen J. (2009). Allogrooming in cattle: Relationships between social preferences, feeding displacements and social dominance. Appl. Anim. Behav. Sci..

[47] Gutmann A.K., Špinka M., Winckler C. (2015). Long-term familiarity creates preferred social partners in dairy cows. Appl. Anim. Behav. Sci..

[48] Windschnurer I., Schmied C., Boivin X., Waiblinger S., Forkman B., Keeling L. (2009). Assessment of Human-Animal Relationships in Dairy Cows. Welfare Quality^®^ Reports.

[49] Napolitano F., Serrapica F., Braghieri A., Masucci F., Sabia E., De Rosa G. (2019). Human-Animal Interactions in Dairy Buffalo Farms. Animals.

[50] Windschnurer I., Schmied C., Boivin X., Waiblinger S. (2008). Reliability and inter-test relationship of tests for on-farm assessment of dairy cows’ relationship to humans. Appl. Anim. Behav. Sci..

[51] Waiblinger S., Menke C., Coleman G. (2002). The relationship between attitudes, personal characteristics and behaviour of stockpeople and subsequent behaviour and production of dairy cows. Appl. Anim. Behav. Sci..

[52] Westerath H.S., Laister S., Winckler C., Knierim U. (2009). Exploration as an indicator of good welfare in beef bulls: An attempt to develop a test for on-farm assessment. Appl. Anim. Behav. Sci..

[53] Mintline E.M., Wood S.L., de Passillé A.M., Rushen J., Tucker C.B. (2012). Assessing calf play behavior in an arena test. Appl. Anim. Behav. Sci..

[54] De Rosa G., Grasso F., Braghieri A., Bilancione A., Di Francia A., Napolitano F. (2009). Behavior and milk production of buffalo cows as affected by housing system. J. Dairy Sci..

[55] Ninomiya S. (2019). Grooming Device Effects on Behaviour and Welfare of Japanese Black Fattening Cattle. Animals.

[56] Westerath H.S., Gygax L., Hillmann E. (2014). Are special feed and being brushed judged as positive by calves?. Appl. Anim. Behav. Sci..

[57] Favreau-Peigné A., Baumont R., Ginane C. (2013). Food sensory characteristics: Their unconsidered roles in the feeding behaviour of domestic ruminants. Animal.

[58] Manteca X., Villalba J.J., Atwood S.B., Dziba L., Provenza F.D. (2008). Is dietary choice important to animal welfare?. J. Vet. Behav. Clin. Appl. Res..

[59] Rutter S.M. (2010). Review: Grazing preferences in sheep and cattle: Implications for production, the environment and animal welfare. Can. J. Anim. Sci..

[60] De Rosa G., Napolitano F., Marino V., Bordi A. (1995). Induction of conditioned taste aversion in goats. Small Rumin. Res..

[61] Provenza F. (1995). Postingestive Feedback as an Elementary Determinant of Food Preference and Intake in Ruminants. J. Range Manag..

[62] Catanese F., Obelar M., Villalba J.J., Distel R.A. (2013). The importance of diet choice on stress-related responses by lambs. Appl. Anim. Behav. Sci..

[63] Dubé L., LeBel J.L., Lu J. (2005). Affect asymmetry and comfort food consumption. Physiol. Behav..

[64] Lin J.Y., Amodeo L.R., Arthurs J., Reilly S. (2012). Taste neophobia and palatability: The pleasure of drinking. Physiol. Behav..

[65] Webb L.E., Engel B., Berends H., van Reenen C.G., Gerrits W.J.J., de Boer I.J.M., Bokkers E.A.M. (2014). What do calves choose to eat and how do preferences affect behaviour?. Appl. Anim. Behav. Sci..

[66] Meagher R.K., Weary D.M., von Keyserlingk M.A.G. (2017). Some like it varied: Individual differences in preference for feed variety in dairy heifers. Appl. Anim. Behav. Sci..

[67] Atwood S.B., Provenza F.D., Wiedmeier R.D., Banner R.E. (2001). Influence of free-choice vs mixed-ration diets on food intake and performance of fattening calves. J. Anim. Sci..

[68] Napolitano F., Knierim U., Grass F., De Rosa G. (2010). Positive indicators of cattle welfare and their applicability to on-farm protocols. Ital. J. Anim. Sci..

[69] Miranda-de la Lama G.C., Mattiello S. (2010). The importance of social behaviour for goat welfare in livestock farming. Small Rumin. Res..

[70] Gautrais J., Michelena P., Sibbald A., Bon R., Deneubourg J.L. (2007). Allelomimetic synchronization in Merino sheep. Anim. Behav..

[71] Bouissou M.F., Boissy A., Le Neindre P., Veissier I., Keeling L., Gonyou H. (2001). The social behaviour of cattle. Social Behaviour in Farm Animals.

[72] Dávid-Barrett T., Dunbar R.I.M. (2012). Cooperation, behavioural synchrony and status in social networks. J. Theor. Biol..

[73] Stoye S., Porter M.A., Stamp Dawkins M. (2012). Synchronized lying in cattle in relation to time of day. Livest. Sci..

[74] Muñoz-Osorio G.A., Aguilar-Caballero A.J., Cámara-Sarmiento R. (2019). Influencia del tipo de alojamiento sobre el comportamiento productivo y bienestar de corderos en sistemas de engorda intensivos. Trop. Subtrop. Agroecosyst..

[75] Petherick J.C., Phillips C.J.C. (2009). Space allowances for confined livestock and their determination from allometric principles. Appl. Anim. Behav. Sci..

[76] Mandel R., Whay H.R., Klement E., Nicol C.J. (2016). Invited review: Environmental enrichment of dairy cows and calves in indoor housing. J. Dairy Sci..

[77] Krohn C.C., Munksgaard L. (1993). Krohn & Munksgaard, 1993_lying in cattle.pdf. Appl. Anim. Behav. Sci..

[78] Lidfors L. (1989). The use of getting up and lying down movements in the evaluation of cattle environments. Vet. Res. Commun..

[79] Jensen M.B., Pedersen L.J., Munksgaard L. (2005). The effect of reward duration on demand functions for rest in dairy heifers and lying requirements as measured by demand functions. Appl. Anim. Behav. Sci..

[80] Hansen I. (2015). Behavioural indicators of sheep and goat welfare in organic and conventional Norwegian farms. Acta Agric. Scand. A Anim. Sci..

[81] Richmond S.E., Wemelsfelder F., de Heredia I.B., Ruiz R., Canali E., Dwyer C.M. (2017). Evaluation of Animal-Based Indicators to Be Used in a Welfare Assessment Protocol for Sheep. Front. Vet. Sci..

[82] Phillips C.J.C. (2002). Cattle Behaviour and Welfare.

[83] Napolitano F., Pacelli C., De Rosa G., Braghieri A., Girolami A. (2005). Sustainability and welfare of Podolian cattle. Livest. Prod. Sci..

[84] Mattiello S., Battini M., Andreoli E., Barbieri S. (2011). Short communication: Breed differences affecting dairy cattle welfare in traditional alpine tie-stall husbandry systems. J. Dairy Sci..

[85] Franco N.H., Magalhães-Sant’Ana M., Olsson I.A.S., Appleby M., Sandøe P., Weary D. (2014). Welfare and quantity of life. Dilemmas in Animal Welfare.

[86] Can E., Vieira A., Battini M., Mattiello S., Stilwell G. (2017). Consistency over time of animal-based welfare indicators as a further step for developing a welfare assessment monitoring scheme: The case of the Animal Welfare Indicators protocol for dairy goats. J. Dairy Sci..

[87] Phythian C.J., Cripps P.J., Michalopoulou E., Jones P.H., Grove-White D., Clarkson M.J., Winter A.C., Stubbings L.A., Duncan J.S. (2012). Reliability of indicators of sheep welfare assessed by a group observation method. Vet. J..

[88] Mellor D.J. (2015). Positive animal welfare states and encouraging environment-focused and animal-to-animal interactive behaviours. N. Z. Vet. J..

[89] Krachun C., Rushen J., de Passillé A.M. (2010). Play behaviour in dairy calves is reduced by weaning and by a low energy intake. Appl. Anim. Behav. Sci..

[90] Thornton P.D., Waterman-Pearson A.E. (2002). Behavioural responses to castration in lambs. Anim. Welf..

[91] Burghardt G. (2005). The genesis of animal play. Nature.

[92] Boissy A., Manteuffel G., Jensen M.B., Moe R.O., Spruijt B., Keeling L.J., Winckler C., Forkman B., Dimitrov I., Langbein J. (2007). Assessment of positive emotions in animals to improve their welfare. Physiol. Behav..

[93] Valníčková B., Stěhulová I., Šárová R., Špinka M. (2015). The effect of age at separation from the dam and presence of social companions on play behavior and weight gain in dairy calves. J. Dairy Sci..

[94] Loberg J., Telezhenko E., Bergsten C., Lidfors L. (2004). Behaviour and claw health in tied dairy cows with varying access to exercise in an outdoor paddock. Appl. Anim. Behav. Sci..

[95] Anderson C., Yngvesson J., Boissy A., Uvnäs-Moberg K., Lidfors L. (2015). Behavioural expression of positive anticipation for food or opportunity to play in lambs. Behav. Process..

[96] Moe R.O., Nordgreen J., Janczak A.M., Spruijt B.M., Zanella A.J., Bakken M. (2009). Trace classical conditioning as an approach to the study of reward-related behaviour in laying hens: A methodological study. Appl. Anim. Behav. Sci..

[97] Held S.D.E., Špinka M. (2011). Animal play and animal welfare. Anim. Behav..

[98] Gygax L., Hillmann E. (2018). “Naturalness” and Its Relation to Animal Welfare from an Ethological Perspective. Agriculture.

[99] Mattiello S., Ferrante V., Verga M., Gottardo F., Andrighetto I., Canali E., Caniatti M., Cozzi G. (2016). The provision of solid feeds to veal calves: II. Behavior, physiology, and abomasal damage1. J. Anim. Sci..

[100] Napolitano F., Annicchiarico G., Caroprese M., De Rosa G., Taibi L., Sevi A. (2003). Lambs prevented from suckling their mothers display behavioral, immune and endocrine disturbances. Physiol. Behav..

[101] Arnold G.W., Dudzinski M.L., Arnold G.W., Dudzinski M.L. (1978). Social organization and animal dispersion. Ethology of Free-Ranging Domestic Animals.

[102] Arnott G., Ferris C.P., O’connell N.E. (2017). Review: Welfare of dairy cows in continuously housed and pasture-based production systems. Animal.

[103] Sato S., Tarumizu K., Hatae K. (1993). The influence of social factors on allogrooming in cows. Appl. Anim. Behav. Sci..

[104] Sato S., Sako S., Maeda A. (1991). Social licking patterns in cattle (Bos taurus): Influence of environmental and social factors. Appl. Anim. Behav. Sci..

[105] Baxter E.M., Mulligan J., Hall S.A., Donbavand J.E., Palme R., Aldujaili E., Zanella A.J., Dwyer C.M. (2016). Positive and negative gestational handling influences placental traits and mother-offspring behavior in dairy goats. Physiol. Behav..

[106] Laister S., Stockinger B., Regner A.M., Zenger K., Knierim U., Winckler C. (2011). Social licking in dairy cattle-Effects on heart rate in performers and receivers. Appl. Anim. Behav. Sci..

[107] Wood M.T. (1977). Social grooming patterns in two herds of monozygotic twin dairy cows. Anim. Behav..

[108] Sato S. (1984). Social licking pattern and its relationships to social dominance and live weight gain in weaned calves. Appl. Anim. Behav. Sci..

[109] Tresoldi G., Weary D.M., Filho L.C.P.M., von Keyserlingk M.A.G. (2015). Social licking in pregnant dairy heifers. Animals.

[110] Krohn C.C. (1994). Behaviour of dairy cows kept in extensive(loose housing/pasture) or intensive (tie stall) environments. III. Grooming, exploration and abnormal behaviour. Appl. Anim. Behav. Sci..

[111] Jensen M.B., Herskin M.S., Thomsen P.T., Forkman B., Houe H. (2015). Preferences of lame cows for type of surface and level of social contact in hospital pens. J. Dairy Sci..

[112] Mooring M.S., Gavazzi A.J., Hart B.L. (1998). Effects of castration on grooming in goats. Physiol. Behav..

[113] Kakuma Y., Takeuchi Y., Mori Y., Hart B.L. (2003). Hormonal control of grooming behavior in domestic goats. Physiol. Behav..

[114] Hart B.L., Pryor P.A. (2004). Developmental and hair-coat determinants of grooming behaviour in goats and sheep. Anim. Behav..

[115] Mooring M.S., Hart B.L., Fitzpatrick T.A., Reisig D.D., Nishihira T.T., Fraser I.C., Benjamin J.E. (2006). Grooming in desert bighorn sheep (*Ovis canadensis mexicana*) and the ghost of parasites past. Behav. Ecol..

[116] Wilson L.L., Terosky T.L., Stull C.L., Stricklin W.R. (1999). Effects of Individual Housing Design and Size on Behavior and Stress Indicators of Special-Fed Holstein Veal Calves. J. Anim. Sci..

[117] Rushen J., de Passillé A.M.B. (1992). The scientific assessment of the impact of housing on animal welfare: A critical review. Can. J. Anim. Sci..

[118] Broom D.M., Metz J.M., Groenestein C.M. (1991). Needs and welfare of housed calves. New Trends in Veal Calf Production.

[119] McConnachie E., Smid A.M.C., Thompson A.J., Weary D.M., Gaworski M.A., Von Keyserlingk M.A.G. (2018). Cows are highly motivated to access a grooming substrate. Biol. Lett..

[120] Mandel R., Whay H.R., Nicol C.J., Klement E. (2013). The effect of food location, heat load, and intrusive medical procedures on brushing activity in dairy cows. J. Dairy Sci..

[121] De Vries M., Bokkers E.A.M., van Reenen C.G., Engel B., van Schaik G., Dijkstra T., de Boer I.J.M. (2015). Housing and management factors associated with indicators of dairy cattle welfare. Prev. Vet. Med..

[122] Bertenshaw C., Rowlinson P., Edge H., Douglas S., Shiel R. (2008). The effect of different degrees of “positive” human-animal interaction during rearing on the welfare and subsequent production of commercial dairy heifers. Appl. Anim. Behav. Sci..

[123] Sabia E., Napolitano F., De Rosa G., Terzano G.M., Barile V.L., Braghieri A., Pacelli C. (2014). Efficiency to reach age of puberty and behaviour of buffalo heifers (*Bubalus bubalis*) kept on pasture or in confinement. Animal.

[124] Hemsworth P.H. (2003). Human-animal interactions in livestock production. Appl. Anim. Behav. Sci..

[125] Breuer K., Hemsworth P., Barnett J., Matthews L., Coleman G. (2000). Behavioural response to humans and the productivity of commercial dairy cows. Appl. Anim. Behav. Sci..

[126] Lensink B.J., Fernandez X., Boivin X., Pradel P. (2000). The impact of gentle contacts on ease of handling, welfare, and. J. Anim. Sci..

[127] Rushen J., de Passillé A.M.B., Munksgaard L. (2010). Fear of People by Cows and Effects on Milk Yield, Behavior, and Heart Rate at Milking. J. Dairy Sci..

[128] Hemsworth P.H., Coleman G.J., Barnett J.L., Borg S. (2000). Relationships between human-animal interactions and productivity of commercial dairy cows. J. Anim. Sci..

[129] Lürzel S., Münsch C., Windschnurer I., Futschik A., Palme R., Waiblinger S. (2015). The influence of gentle interactions on avoidance distance towards humans, weight gain and physiological parameters in group-housed dairy calves. Appl. Anim. Behav. Sci..

[130] Serrapica M., Boivin X., Coulon M., Braghieri A., Napolitano F. (2017). Positive perception of human stroking by lambs: Qualitative behaviour assessment confirms previous interpretation of quantitative data. Appl. Anim. Behav. Sci..

[131] Coulon M., Nowak R., Peyrat J., Chandèze H., Boissy A., Boivin X. (2015). Do Lambs Perceive Regular Human Stroking as Pleasant? Behavior and Heart Rate Variability Analyses. PLoS ONE.

[132] Guesdon V., Nowak R., Meurisse M., Boivin X., Cornilleau F., Chaillou E., Lévy F. (2016). Behavioral evidence of heterospecific bonding between the lamb and the human caregiver and mapping of associated brain network. Psychoneuroendocrinology.

[133] Ellingsen K., Coleman G.J., Lund V., Mejdell C.M. (2014). Using qualitative behaviour assessment to explore the link between stockperson behaviour and dairy calf behaviour. Appl. Anim. Behav. Sci..

[134] Winckler C., Brinkmann J., Glatz J. (2007). Long-term consistency of selected animal-related welfare parameters in dairy farms. Anim. Welf..

[135] Waiblinger S., Menke C., Fölsch D.W. (2003). Influences on the avoidance and approach behaviour of dairy cows towards humans on 35 farms. Appl. Anim. Behav. Sci..

[136] Battini M., Barbieri S., Waiblinger S., Mattiello S. (2016). Validity and feasibility of Human-Animal Relationship tests for on-farm welfare assessment in dairy goats. Appl. Anim. Behav. Sci..

[137] Tripaldi C., De Rosa G., Grasso F., Terzano G.M., Napolitano F. (2004). Housing system and welfare of buffalo (*Bubalus bubalis*) cows. Anim. Sci..

[138] Napolitano F., Pacelli C., Grasso F., Braghieri A., De Rosa G. (2013). The behaviour and welfare of buffaloes (*Bubalus bubalis*) in modern dairy enterprises. Animal.

[139] Aggarwal A., Singh M. (2008). Changes in skin and rectal temperature in lactating buffaloes provided with showers and wallowing during hot-dry season. Trop. Anim. Health Prod..

[140] Bracke M.B.M. (2011). Review of wallowing in pigs: Description of the behaviour and its motivational basis. Appl. Anim. Behav. Sci..

[141] Hafez E.S.E., Cairns R.B., Hulet C.V., Scott J.P., Hafez E.S.E. (1969). The behaviour of sheep and goats. The Behaviour of Domestic Animals.

[142] Aschwanden J., Gygax L., Wechsler B., Keil N.M. (2009). Loose housing of small goat groups: Influence of visual cover and elevated levels on feeding, resting and agonistic behaviour. Appl. Anim. Behav. Sci..

[143] Pickup H.E., Dwyer C.M. (2011). Breed differences in the expression of maternal care at parturition persist throughout the lactation period in sheep. Appl. Anim. Behav. Sci..

[144] Nowak R., Boivin X. (2015). Filial attachment in sheep: Similarities and differences between ewe-lamb and human-lamb relationships. Appl. Anim. Behav. Sci..

[145] Muir E., Donbavand J., Dwyer C.M. Salivary oxytocin is associated with ewe-lamb contact but not suckling in lactating ewes. Proceedings of the 53rd Congress of the International Society of Applied Ethology.

[146] Panksepp J. (2011). The basic emotional circuits of mammalian brains: Do animals have affective lives?. Neurosci. Biobehav. Rev..

[147] Mendl M., Burman O.H.P., Parker R.M.A., Paul E.S. (2009). Cognitive bias as an indicator of animal emotion and welfare: Emerging evidence and underlying mechanisms. Appl. Anim. Behav. Sci..

[148] Baciadonna L., McElligott A.G. (2015). The use of judgement bias to assess welfare in farm livestock. Anim. Welf..

[149] Roelofs S., Boleij H., Nordquist R.E., van der Staay F.J. (2016). Making Decisions under Ambiguity: Judgment Bias Tasks for Assessing Emotional State in Animals. Front. Behav. Neurosci..

[150] Brajon S., Laforest J.P., Schmitt O., Devillers N. (2015). The way humans behave modulates the emotional state of piglets. PLoS ONE.

[151] Zidar J., Campderrich I., Jansson E., Wichman A., Winberg S., Keeling L., Løvlie H. (2018). Environmental complexity buffers against stress-induced negative judgement bias in female chickens. Sci. Rep..

[152] Doyle R.E., Fisher A.D., Hinch G.N., Boissy A., Lee C. (2010). Release from restraint generates a positive judgement bias in sheep. Appl. Anim. Behav. Sci..

[153] Sanger M.E., Doyle R.E., Hinch G.N., Lee C. (2011). Sheep exhibit a positive judgement bias and stress-induced hyperthermia following shearing. Appl. Anim. Behav. Sci..

[154] Crump A., Arnott G., Bethell E.J. (2018). Affect-driven attention biases as animal welfare indicators: Review and methods. Animals.

[155] Lee C., Verbeek E., Doyle R., Bateson M. (2016). Attention bias to threat indicates anxiety differences in sheep. Biol. Lett..

[156] Lee C., Cafe L.M., Robinson S.L., Doyle R.E., Lea J.M., Small A.H., Colditz I.G. (2018). Anxiety influences attention bias but not flight speed and crush score in beef cattle. Appl. Anim. Behav. Sci..

[157] Whittaker A.L., Marsh L.E. (2019). The role of behavioural assessment in determining ‘positive’ affective states in animals. CAB Rev. Perspect. Agric. Vet. Sci. Nutr. Nat. Resour..

[158] Reefmann N., Bütikofer Kaszàs F., Wechsler B., Gygax L. (2009). Ear and tail postures as indicators of emotional valence in sheep. Appl. Anim. Behav. Sci..

[159] Mendl M., Burman O.H.P., Paul E.S. (2010). An integrative and functional framework for the study of animal emotion and mood. Proc. R. Soc. B Biol. Sci..

[160] Wemelsfelder F., Hunter T.E.A., Mendl M.T., Lawrence A.B. (2001). Assessing the “whole animal”: A free choice profiling approach. Anim. Behav..

[161] Napolitano F., De Rosa G., Braghieri A., Grasso F., Bordi A., Wemelsfelder F. (2008). The qualitative assessment of responsiveness to environmental challenge in horses and ponies. Appl. Anim. Behav. Sci..

[162] Wemelsfelder F., Millard F., De Rosa G., Napolitano F., Forkman B., Keeling L. (2009). Qualitative behaviour assessment. Welfare Quality^®^ Report No. 11—Assessment of Animal Welfare Measures for Dairy Cattle, Beef Bulls and Veal Calves.

[163] Phythian C., Michalopoulou E., Duncan J., Wemelsfelder F. (2013). Inter-observer reliability of Qualitative Behavioural Assessments of sheep. Appl. Anim. Behav. Sci..

[164] Phythian C.J., Michalopoulou E., Cripps P.J., Duncan J.S., Wemelsfelder F. (2016). On-farm qualitative behaviour assessment in sheep: Repeated measurements across time, and association with physical indicators of flock health and welfare. Appl. Anim. Behav. Sci..

[165] Sant’Anna A.C., Paranhos da Costa M.J.R. (2013). Validity and feasibility of qualitative behavior assessment for the evaluation of Nellore cattle temperament. Livest. Sci..

[166] Grosso L., Battini M., Wemelsfelder F., Barbieri S., Minero M., Dalla Costa E., Mattiello S. (2016). On-farm Qualitative Behaviour Assessment of dairy goats in different housing conditions. Appl. Anim. Behav. Sci..

[167] Proctor H.S., Carder G. (2015). Measuring positive emotions in cows: Do visible eye whites tell us anything?. Physiol. Behav..

[168] Sandem A.I., Janczak A.M., Salte R., Braastad B.O. (2006). The use of diazepam as a pharmacological validation of eye white as an indicator of emotional state in dairy cows. Appl. Anim. Behav. Sci..

[169] Sandem A.I., Braastad B.O., Bakken M. (2006). Behaviour and percentage eye-white in cows waiting to be fed concentrate—A brief report. Appl. Anim. Behav. Sci..

[170] Sandem A.-I., Braastad B.O. (2005). Effects of cow-calf separation on visible eye white and behaviour in dairy cows—A brief report. Appl. Anim. Behav. Sci..

[171] Lambert (Proctor) H.S., Carder G. (2017). Looking into the eyes of a cow: Can eye whites be used as a measure of emotional state?. Appl. Anim. Behav. Sci..

[172] Tamioso P.R., Rucinque D.S., Taconeli C.A., da Silva G.P., Molento C.F.M. (2017). Behavior and body surface temperature as welfare indicators in selected sheep regularly brushed by a familiar observer. J. Vet. Behav. Clin. Appl. Res..

[173] Tamioso P.R., Maiolino Molento C.F., Boivin X., Chandèze H., Andanson S., Delval É., Hazard D., da Silva G.P., Taconeli C.A., Boissy A. (2018). Inducing positive emotions: Behavioural and cardiac responses to human and brushing in ewes selected for high vs low social reactivity. Appl. Anim. Behav. Sci..

[174] Reefmann N., Bütikofer F., Wechsler B., Gygax L. (2009). Physiological expression of emotional reactions in sheep. Physiol. Behav..

[175] Reefmann N., Wechsler B., Gygax L. (2009). Behavioural and physiological assessment of positive and negative emotion in sheep. Anim. Behav..

[176] Battini M., Agostini A., Mattiello S. (2019). Understanding cows’ emotions on farm: Are eye white and ear posture reliable indicators?. Animals.

[177] Bellegarde L.G.A., Haskell M.J., Duvaux-ponter C., Weiss A., Boissy A., Erhard H.W. (2017). Face-based perception of emotions in dairy goats. Appl. Anim. Behav. Sci..

[178] Proctor H.S., Carder G. (2014). Can ear postures reliably measure the positive emotional state of cows?. Appl. Anim. Behav. Sci..

[179] Schmied C., Waiblinger S., Scharl T., Leisch F., Boivin X. (2008). Stroking of different body regions by a human: Effects on behaviour and heart rate of dairy cows. Appl. Anim. Behav. Sci..

[180] Boissy A., Aubert A., Greiveldinger L., Delval E., Veissier I. (2011). Cognitive sciences to relate ear postures to emotions in sheep. Anim. Welf..

[181] Briefer E.F., Tettamanti F., McElligott A.G. (2015). Emotions in goats: Mapping physiological, behavioural and vocal profiles. Anim. Behav..

[182] De Oliveira D., Keeling L.J. (2018). Routine activities and emotion in the life of dairy cows: Integrating body language into an affective state framework. PLoS ONE.

[183] Padilla de la Torre M., Briefer E.F., Reader T., McElligott A.G. (2015). Acoustic analysis of cattle (*Bos taurus*) mother-offspring contact calls from a source-filter theory perspective. Appl. Anim. Behav. Sci..

[184] Fisher A., Matthews L., Keeling L., Gonyou H. (2001). The social behavior of sheep. Social Behavior in Farm Animals.

